# Supplementary material for: The distribution of functional N-cycle related genes and ammonia and nitrate nitrogen in soil profiles fertilized with mineral and organic N fertilizer
Source: PLoS One. 2020 Jun 2;15(6):e0228364. doi: 10.1371/journal.pone.0228364 (PMC7266355; doi:10.1371/journal.pone.0228364)

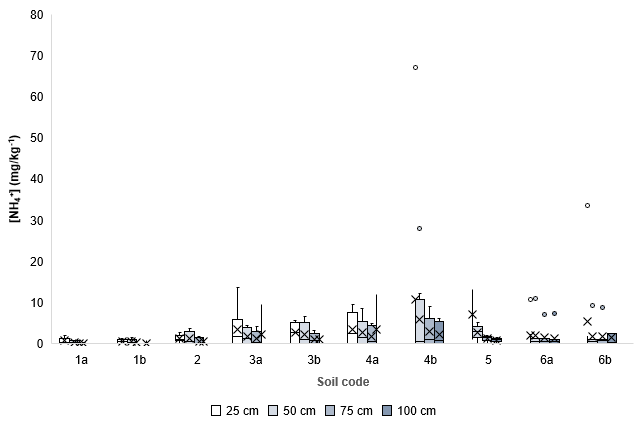


***b***

***a***

**S1 Fig. Ammonium (*a*) and phosphate (*b*) concentration in soil.** For each experiment, divided for depth classes, the box plot shows minimum and maximum values (bars), the first and the third quartile (boxes), the median (lines inside boxes) and the average (crosses); *n*=248.


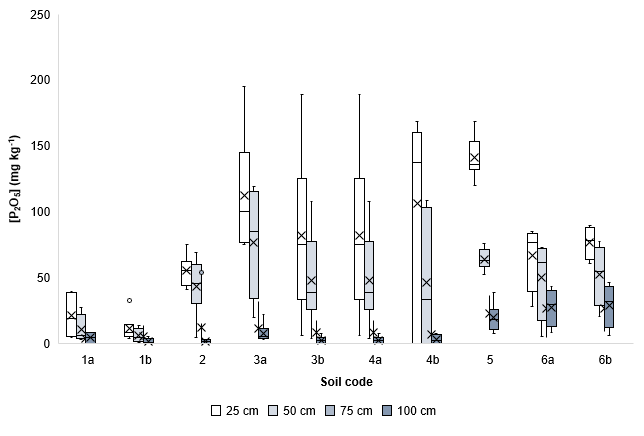

Supplement: S1 Fig — Ammonium (a) and phosphate (b) concentration in soil. For each experiment, divided for depth classes, the box plot shows minimum and maximum values (bars), the first and the third quartile (boxes), the median (lines inside boxes) and the average (crosses); n = 248. (DOCX) [file pone.0228364.s001.docx]
