# Supplementary material for: The distribution of functional N-cycle related genes and ammonia and nitrate nitrogen in soil profiles fertilized with mineral and organic N fertilizer
Source: PLoS One. 2020 Jun 2;15(6):e0228364. doi: 10.1371/journal.pone.0228364 (PMC7266355; doi:10.1371/journal.pone.0228364)
Supplement: S1 Table — In the Table are reported the agronomic data of all the experiments made. (DOCX) [file pone.0228364.s002.docx]

**S1 Table. Soil studied and soil agronomic management.** In the Table are reported the agronomic data of all the experiments made.

| Soil Code | Year | Crops | Fertilizer | Irrigation | Crop management | Time of fertilization | Total N supplied (kg ha^-1^) | Crop Harvest date | Field area (Ha) |
| --- | --- | --- | --- | --- | --- | --- | --- | --- | --- |
| 1a | 2014 | Maize | Cattle slurry | Yes | Tillage | 16/05/2014  12/06/2014 | 117  140 | 07/10/2014 | 2.95 |
| 1b |  | Maize – Cover crop | Urea | Yes | Tillage | 31/03/2014  02/07/2014 | 116  164 | 17/10/2014 | 4.41 |
| 2 |  | Barley – Silage maize | Digestate liquid fraction | No | Tillage | 18/04/2014  13/05/2014 | 94  59 | 11/09/2014 | 4.76 |
| 3a | 2015 | Maize | Digestate liquid fraction | Yes | Tillage | 13/05/2015  15/06/2015 | 178  153 | 31/08/2015 | 2.63 |
| 3b |  | Maize | Pig slurry | Yes | Tillage | 13/05/2015  15/06/2015 | 300  104 | 31/08/2015 | 1.73 |
| 4a | 2016 | Maize | Digestate liquid fraction + Urea | Yes | Tillage | 06/04/2016  07/06/2016 | 245  138 | 25/08/2016 | 2.63 |
| 4b |  | Maize | Pig slurry + Urea | Yes | Tillage | 06/04/2016  07/06/2016 | 287  138 | 25/08/2016 | 1.73 |
| 5 |  | Maize | Digestate | Yes | Tillage | 08/06/2016  13/06/2016 | 222  47 | 25/09/2016 | 3.01 |
| 6a |  | Maize | Digestate | Yes | Tillage | 24/06/2016  25/07/2016 | 269  110 | 06/10/2016 | 3.5 |
| 6b |  | Maize | Digestate + Urea | Yes | Tillage | 24/06/2016  25/07/2016 | 269  184 | 06/10/2016 | 3.41 |
| 7 (4a + extra N) |  | Maize | Digestate liquid fraction + Urea + Pig Slurry | Yes | Tillage | 06/04/2016  07/06/2016  03/10/2016 | 245  138  860 | 25/08/2016 | 2.63 |
| 8 |  | Maize | Digestate + Urea + Pig slurry | Yes | Tillage | 10/04/2016  28/07/2016  24/10/2016 | 270  620  580 | 22/08/2016 | 4.34 |
