# Supplementary material for: The distribution of functional N-cycle related genes and ammonia and nitrate nitrogen in soil profiles fertilized with mineral and organic N fertilizer
Source: PLoS One. 2020 Jun 2;15(6):e0228364. doi: 10.1371/journal.pone.0228364 (PMC7266355; doi:10.1371/journal.pone.0228364)
Supplement: S2 Table — The months of the year in which at least one sampling was carried out for the corresponding Soil are marked in grey. (DOCX) [file pone.0228364.s003.docx]

**S2 Table. Sampling periods.** The months of the year in which at least one sampling was carried out for the corresponding Soil are marked in grey.

| **Soil Code** | **Months** | | | | | | | | | | | |
| --- | --- | --- | --- | --- | --- | --- | --- | --- | --- | --- | --- | --- |
|  | **Jan** | **Feb** | **Mar** | **Apr** | **May** | **Jun** | **Jul** | **Aug** | **Sep** | **Oct** | **Nov** | **Dec** |
| **1a** |  |  |  |  |  |  |  |  |  |  |  |  |
| **1b** |  |  |  |  |  |  |  |  |  |  |  |  |
| **2** |  |  |  |  |  |  |  |  |  |  |  |  |
| **3a** |  |  |  |  |  |  |  |  |  |  |  |  |
| **3b** |  |  |  |  |  |  |  |  |  |  |  |  |
| **4a** |  |  |  |  |  |  |  |  |  |  |  |  |
| **4b** |  |  |  |  |  |  |  |  |  |  |  |  |
| **5** |  |  |  |  |  |  |  |  |  |  |  |  |
| **6a** |  |  |  |  |  |  |  |  |  |  |  |  |
| **6b** |  |  |  |  |  |  |  |  |  |  |  |  |
| **7** |  |  |  |  |  |  |  |  |  |  |  |  |
| **8** |  |  |  |  |  |  |  |  |  |  |  |  |
