# Supplementary material for: The distribution of functional N-cycle related genes and ammonia and nitrate nitrogen in soil profiles fertilized with mineral and organic N fertilizer
Source: PLoS One. 2020 Jun 2;15(6):e0228364. doi: 10.1371/journal.pone.0228364 (PMC7266355; doi:10.1371/journal.pone.0228364)
Supplement: S3 Table — For each primer are shown name, nucleotide sequence, target gene and reference. N = undefined nucleotide. (DOCX) [file pone.0228364.s004.docx]

**S3 Table. Primers used in the quantification of nitrogen related microorganisms in soil.** For each primer are shown name, nucleotide sequence, target gene and reference. N = undefined nucleotide.

| **Primer Name** | **Sequence 5’ -> 3’** | **Gene Name** | **GenBank ID** | **References** |
| --- | --- | --- | --- | --- |
| **Arch-amoAF** | NTAATGGTCTGGCTTAGACG | Archaeal *amoA* | MH318568.1 | Francis et al., 2005^1^ |
| **Arch-amoAR** | GCGGCCATCCATCTGTATGT | Archaeal *amoA* | MH318568.1 | Francis et al., 2005^1^ |
| **amoA1F** | GGGGTTTCTACTGGTGGT | Bacterial *amoA* | MG913925.1 | Rotthauwe et al., 1997^2^ |
| **amoA2R** | CCCCTCNGNAAAGCCTTCTTC | Bacterial *amoA* | MG913925.1 | Rotthauwe et al., 1997^2^ |
| **nosZF** | CGNTGTTCNTCGACAGCCAG | *nosZ* | CP000490.1 | Rösch et al., 2002^3^ |
| **nosZR** | CATGTGCAGNGCNTGGCAGAA | *nosZ* | CP000490.1 | Rösch et al., 2002^3^ |
| **nirK876F** | ATNGGCGGNCANGGCGA | *nirK* | KJ147768.1 | Henry et al., 2004^4^ |
| **nirK1040** | NGCCTCGATCAGNTTNTGGTT | *nirK* | KJ147768.1 | Henry et al., 2004^4^ |
| **nifHF** | AAAGGNGGNATCGGNAANTCCACCAC | *nifH* | KC445685.1 | Rösch et al., 2002^3^ |
| **nifHR** | TTGTTNGCNGCNTACATNGCCATCAT | *nifH* | KC445685.1 | Rösch et al., 2002^3^ |
