# Supplementary material for: The distribution of functional N-cycle related genes and ammonia and nitrate nitrogen in soil profiles fertilized with mineral and organic N fertilizer
Source: PLoS One. 2020 Jun 2;15(6):e0228364. doi: 10.1371/journal.pone.0228364 (PMC7266355; doi:10.1371/journal.pone.0228364)
Supplement: S4 Table — Their concentration (average ± standard deviation) in the analysed soils. (DOCX) [file pone.0228364.s005.docx]

**S4 Table. Nitrate, ammonium.** Their concentration (average ± standard deviation) in the analysed soils.

| **Soil Code** | **Depth (cm)** | **NO_3_^-^ (mg**·**kg^-1^)** | **NH_4_^+^ (mg**·**kg^-1^)** |
| --- | --- | --- | --- |
| 1a | 0 – 25 | 11.30 (±15.35; n=5) | 0.70 (±0.74; n=5) |
|  | 25 – 50 | 6.60 (±4.52; n=5) | 0.42 (±0.42; n=5) |
|  | 50 – 75 | 3.41 (±1.78; n=5) | 0.08 (±0.12; n=5) |
|  | 75 – 100 | 1.53 (±1.47; n=5) | 0.05 (±0.11; n=5) |
| 1b | 0 – 25 | 20.12 (±21.02; n=6) | 0.55 (±0.61; n=6) |
|  | 25 – 50 | 8.17 (±4.81; n=6) | 0.51 (±0.61; n=6) |
|  | 50 – 75 | 4.10 (±1.48; n=6) | 0.25 (±0.36; n=5) |
|  | 75 – 100 | 1.67 (±1.71; n=6) | 0.18 (±0.41; n=5) |
| 2 | 0 – 25 | 19.41 (±12.08; n=8) | 1.29 (±0.87; n=8) |
|  | 25 – 50 | 11.55 (±4.5; n=8) | 1.34 (±1.57; n=8) |
|  | 50 – 75 | 2.8 (±1.27; n=8) | 0.54 (±0.8; n=7) |
|  | 75 – 100 | 1.14 (±0.56; n=5) | 0.45 (±0.69; n=4) |
| 3a | 0 – 25 | 13.53 (±6.26; n=6) | 3.4 (±5.23; n=6) |
|  | 25 – 50 | 5.63 (±1.97; n=6) | 1.75 (±1.92; n=6) |
|  | 50 – 75 | 2.29 (±0.96; n=6) | 1.27 (±1.76; n=6) |
|  | 75 – 100 | 5.08 (±3.96; n=6) | 2.31 (±3.79; n=6) |
| 3b | 0 – 25 | 10.69 (±11.62; n=6) | 2.72 (±2.48; n=6) |
|  | 25 – 50 | 5.5 (±2.26; n=6) | 2.29 (±2.86; n=6) |
|  | 50 – 75 | 4.28 (±5.72; n=6) | 1.17 (±1.38; n=6) |
|  | 75 – 100 | 3.31 (±4.59; n=6) | 1 (±1.07; n=6) |
| 4a | 0 – 25 | 15.32 (±8.86; n=6) | 4.66 (±3.79; n=6) |
|  | 25 – 50 | 11.54 (±6.75; n=6) | 3.72 (±2.29; n=6) |
|  | 50 – 75 | 8.75 (±5.44; n=6) | 2.33 (±3.33; n=6) |
|  | 75 – 100 | 6.01 (±5.44; n=6) | 3.27 (±3.33; n=6) |
| 4b | 0 – 25 | 24.38 (±8.51; n=5) | 1.93 (±2.63; n=5) |
|  | 25 – 50 | 16.37 (±8.86; n=6) | 7.99 (±10.92; n=6) |
|  | 50 – 75 | 11.98 (±8.76; n=6) | 3.85 (±3.73; n=6) |
|  | 75 – 100 | 9.15 (±6.66; n=6) | 2.98 (±2.8; n=6) |
| 5 | 0 – 25 | 20.67 (±6.78; n=7) | 7.09 (±4.26; n=7) |
|  | 25 – 50 | 14.17 (±4.15; n=6) | 2.86 (±1.83; n=6) |
|  | 50 – 75 | 10.15 (±2.2; n=6) | 1.37 (±0.74; n=6) |
|  | 75 – 100 | 8.27 (±2.6; n=6) | 0.91 (±0.5; n=6) |
| 6a | 0 – 25 | 9.5 (±6.2; n=7) | 2.13 (±3.86; n=7) |
|  | 25 – 50 | 5.23 (±2.93; n=7) | 2.01 (±3.97; n=7) |
|  | 50 – 75 | 1.64 (±1.19; n=7) | 1.44 (±2.55; n=7) |
|  | 75 – 100 | 1.64 (±1.19; n=7) | 1.44 (±2.55; n=7) |
| 6b | 0 – 25 | 9.46 (±5.37; n=7) | 5.33 (±12.44; n=7) |
|  | 25 – 50 | 5.36 (±2.3; n=7) | 1.73 (±3.42; n=7) |
|  | 50 – 75 | 2.8 (±1.68; n=7) | 1.67 (±3.22; n=7) |
|  | 75 – 100 | 1.43 (±1.13; n=6) | 1.49 (±2.8; n=6) |
| 7 | 0 – 25 | 39.08 (±46.39; n=7) | 6.75 (±5.54; n=7) |
|  | 25 – 50 | 19.25 (±16.37; n=8) | 3.35 (±2.94; n=8) |
|  | 50 – 75 | 10.13 (±6.29; n=8) | 1.91 (±2.09; n=8) |
|  | 75 – 100 | 6.22 (±4.67; n=8) | 3.74 (±4.36; n=8) |
| 8 | 0 – 25 | 28.02 (±17.92; n=6) | 2.43 (±1.81; n=6) |
|  | 25 – 50 | 28.95 (±11.43; n=6) | 1.31 (±1.29; n=6) |
|  | 50 – 75 | 22.6 (±11.63; n=6) | 0.89 (±0.83; n=6) |
|  | 75 – 100 | 25.61 (±17.43; n=6) | 0.66 (±0.72; n=6) |
