# Supplementary material for: The distribution of functional N-cycle related genes and ammonia and nitrate nitrogen in soil profiles fertilized with mineral and organic N fertilizer
Source: PLoS One. 2020 Jun 2;15(6):e0228364. doi: 10.1371/journal.pone.0228364 (PMC7266355; doi:10.1371/journal.pone.0228364)
Supplement: S5 Table — Average ± standard deviation. (DOCX) [file pone.0228364.s006.docx]

**S5 Table. Gene copies for every microorganism class in the soils analysed**. Average ± standard deviation

| Soil Code | Depth (cm) | | *amoA* Archea (copies·g^-1^) | *amoA* Eubacteria (copies·g^-1^) | *nifH*  (copies·g^-1^) | *nirK* (copies·g^-1^) | *nosZ*  (copies·g^-1^) |
| --- | --- | --- | --- | --- | --- | --- | --- |
| 1a | | 0 – 25 | 4.12E+05  (±1.75E+05; n=5) | 2.04E+05  (±1.62E+05; n=5) | 1.79E+06  (±1.15E+06; n=5) | 2.37E+07  (±1.41E+07; n=5) | 1.11E+06  (±5.38E+05; n=5) |
|  |  | 25 – 50 | 3.99E+05  (±1.31E+05; n=5) | 1.42E+05  (±7.65E+04; n=5) | 1.79E+06  (±8.33E+05; n=5) | 1.97E+07  (±1.33E+07; n=5) | 1.29E+06  (±6.64E+05; n=5) |
|  |  | 50 – 75 | 1.18E+04  (±7.64E+03; n=5) | 0.00E+00  (±0.00E+00; n=5) | 3.10E+04  (±2.20E+04; n=5) | 6.43E+04  (±4.14E+04; n=5) | 3.74E+03  (±2.61E+03; n=5) |
|  |  | 75 – 100 | 4.90E+04 (±4.90E+04; n=5) | 9.19E+05  (±9.19E+05; n=5) | 6.53E+05  (±6.51E+05; n=5) | 6.91E+06  (±6.91E+06; n=5) | 7.80E+05  (±7.80E+05; n=5) |
| 1b | | 0 – 25 | 8.90E+05  (±3.92E+05; n=6) | 5.81E+05  (±2.99E+05; n=6) | 3.42E+06  (±2.30E+06; n=6) | 3.62E+07  (±1.75E+07; n=6) | 1.69E+06  (±8.05E+05; n=6) |
|  |  | 25 – 50 | 1.20E+06  (±7.51E+05; n=6) | 1.56E+06  (±9.40E+05; n=6) | 6.76E+06  (±4.91E+06; n=6) | 4.66E+07  (±3.00E+07; n=6) | 5.09E+06  (±3.77E+06; n=6) |
|  |  | 50 – 75 | 3.71E+05  (±2.87E+05; n=6) | 3.57E+05  (±2.41E+05; n=6) | 9.42E+05  (±6.45E+05; n=6) | 1.01E+07  (±7.69E+06; n=6) | 6.61E+05  (±4.22E+05; n=6) |
|  |  | 75 – 100 | 0.00E+00  (±0.00E+00; n=6) | 0.00E+00  (±0.00E+00; n=6) | 3.66E+02  (±3.66E+02; n=6) | 0.00E+00  (±0.00E+00; n=6) | 0.00E+00  (±0.00E+00; n=6) |
| 2 | | 0 – 25 | 6.85E+06  (±1.79E+06; n=8) | 2.01E+07  (±3.70E+06; n=8) | 1.31E+07  (±2.97E+06; n=8) | 7.81E+07  (±1.40E+07; n=8) | 6.62E+06  (±1.40E+06; n=8) |
|  |  | 25 – 50 | 2.60E+06  (±7.98E+05; n=8) | 8.36E+06  (±9.97E+05; n=8) | 4.66E+06  (±8.01E+05; n=8) | 4.32E+07  (±9.72E+06; n=8) | 3.29E+06  (±5.37E+05; n=8) |
|  |  | 50 – 75 | 1.83E+05  (±5.05E+04; n=8) | 2.08E+06  (±8.36E+05; n=8) | 1.34E+06  (±3.34E+05; n=8) | 9.58E+06  (±2.56E+06; n=8) | 1.23E+06  (±3.33E+05; n=8) |
|  |  | 75 – 100 | 5.52E+04  (±2.74E+04; n=8) | 4.09E+05  (±2.08E+05; n=8) | 4.48E+05  (±1.62E+05; n=8) | 4.66E+06  (±1.97E+06; n=8) | 5.96E+05  (±2.25E+05; n=8) |
| 3a | | 0 – 25 | 1.81E+07  (±1.18E+07; n=6) | 8.43E+07  (±5.33E+07; n=6) | 2.92E+07  (±1.48E+07; n=6) | 5.94E+05  (±2.84E+05; n=6) | 6.26E+07  (±3.71E+07; n=6) |
|  |  | 25 – 50 | 1.92E+07  (±9.80E+06; n=6) | 9.91E+07  (±4.96E+07; n=6) | 3.11E+07  (±1.86E+07; n=6) | 1.03E+06  (±5.12E+05; n=6) | 1.07E+08  (±5.84E+07; n=6) |
|  |  | 50 – 75 | 1.73E+07  (±1.05E+07; n=6) | 3.79E+07  (±1.96E+07; n=6) | 2.47E+07  (±1.44E+07; n=6) | 6.53E+05  (±2.54E+05; n=6) | 5.63E+07  (±3.13E+07; n=6) |
|  |  | 75 – 100 | 4.41E+07  (±4.14E+07; n=6) | 3.72E+07  (±2.09E+07; n=6) | 1.90E+07  (±1.02E+07; n=6) | 8.40E+05  (±5.32E+05; n=6) | 5.15E+07  (±3.71E+07; n=6) |
| 3b | | 0 – 25 | 1.90E+07  (±1.41E+07; n=6) | 7.92E+07  (±4.58E+07; n=6) | 1.96E+07  (±1.12E+07; n=6) | 1.46E+06  (±9.70E+05; n=6) | 1.27E+08  (±9.75E+07; n=6) |
|  |  | 25 – 50 | 3.53E+06  (±3.42E+06; n=6) | 3.16E+07  (±3.00E+07; n=6) | 2.97E+07  (±2.85E+07; n=6) | 3.65E+07  (±3.30E+07; n=6) | 8.28E+05  (±3.65E+05; n=6) |
|  |  | 50 – 75 | 3.11E+05  (±1.77E+05; n=6) | 9.35E+06  (±5.88E+06; n=6) | 5.42E+06  (±2.45E+06; n=6) | 1.17E+06  (±3.77E+05; n=6) | 1.03E+07  (±6.50E+06; n=6) |
|  |  | 75 – 100 | 2.69E+07  (±2.63E+07; n=6) | 1.59E+07  (±1.51E+07; n=6) | 2.21E+07  (±1.39E+07; n=6) | 1.41E+06  (±7.46E+05; n=6) | 8.15E+07  (±5.66E+07; n=6) |
| 4a | | 0 – 25 | 1.11E+09  (±2.08E+09; n=6) | 1.10E+09  (±1.68E+09; n=6) | 5.41E+08  (±5.50E+08; n=6) | 6.68E+08  (±1.01E+09; n=6) | 3.56E+09  (±5.71E+09; n=6) |
|  |  | 25 – 50 | 1.06E+10  (±1.19E+10; n=6) | 2.83E+09  (±3.50E+09; n=6) | 1.79E+09  (±2.47E+09; n=6) | 1.25E+09  (±1.17E+09; n=6) | 1.13E+10  (±1.85E+10; n=6) |
|  |  | 50 – 75 | 2.96E+09  (±4.92E+09; n=6) | 1.47E+09  (±2.32E+09; n=6) | 7.22E+08  (±1.20E+09; n=6) | 1.05E+09  (±1.71E+09; n=6) | 7.25E+09  (±1.52E+10; n=6) |
|  |  | 75 – 100 | 8.89E+07  (±1.54E+08; n=6) | 2.26E+08  (±4.71E+08; n=6) | 2.69E+08  (±5.05E+08; n=6) | 3.41E+08  (±6.29E+08; n=6) | 5.46E+08  (±1.01E+09; n=6) |
| 4b | | 0 – 25 | 3.33E+09  (±3.09E+09; n=5) | 1.77E+09  (±1.66E+09; n=5) | 5.30E+08  (±3.31E+08; n=5) | 1.23E+09  (±1.12E+09; n=5) | 5.17E+09  (±6.48E+09; n=5) |
|  |  | 25 – 50 | 3.80E+09  (±3.24E+09; n=6) | 1.53E+09  (±1.67E+09; n=6) | 1.05E+09  (±1.39E+09; n=6) | 1.34E+09  (±1.08E+09; n=6) | 6.53E+09  (±7.86E+09; n=6) |
|  |  | 50 – 75 | 1.41E+09  (±2.39E+09; n=6) | 5.20E+08  (±9.87E+08; n=6) | 7.37E+08  (±1.06E+09; n=6) | 5.33E+08  (±6.70E+08; n=6) | 2.00E+09  (±2.71E+09; n=6) |
|  |  | 75 – 100 | 4.60E+08  (±1.11E+09; n=6) | 8.57E+07  (±1.97E+08; n=6) | 2.70E+08  (±4.92E+08; n=6) | 1.68E+08  (±3.65E+08; n=6) | 1.23E+09  (±2.14E+09; n=6) |
| 5 | | 0 – 25 | 5.04E+09  (±8.15E+09; n=7) | 1.34E+09  (±1.5E+09; n=7) | 1.81E+08  (±1.09E+08; n=7) | 1.35E+09  (±1.2E+09; n=7) | 4.42E+09  (±6.9E+09; n=7) |
|  |  | 25 – 50 | 9.25E+08  (±6.27E+08; n=6) | 3.60E+08  (±2.91E+08; n=6) | 3.65E+08  (±2.64E+08; n=6) | 5.04E+08  (±3.34E+08; n=6) | 5.85E+08  (±2.89E+08; n=6) |
|  |  | 50 – 75 | 2.86E+09  (±1.32E+09; n=6) | 1.35E+09  (±1.32E+09; n=6) | 2.45E+08  (±1.07E+08; n=6) | 1.11E+09  (±6.05E+08; n=6) | 3.38E+09  (±2.43E+09; n=6) |
|  |  | 75 – 100 | 1.15E+09  (±5.78E+08; n=6) | 5.10E+07  (±3.1E+07; n=6) | 2.88E+08  (±1.66E+08; n=6) | 4.69E+08  (±2.01E+08; n=6) | 7.05E+08  (±3.59E+08; n=6) |
| 6a | | 0 – 25 | 1.07E+08  (±2.44E+07; n=7) | 1.87E+07  (±1.52E+07; n=7) | 3.04E+07  (±1.44E+07; n=7) | 6.64E+06  (±3.99E+06; n=7) | 4.70E+06  (±2.55E+06; n=7) |
|  |  | 25 – 50 | 7.04E+07  (±4.35E+07; n=7) | 3.11E+06  (±2.11E+06; n=7) | 1.28E+07  (±1.06E+07; n=7) | 1.35E+06  (±8.32E+05; n=7) | 1.17E+06  (±8.61E+05; n=7) |
|  |  | 50 – 75 | 1.50E+06  (±1.32E+06; n=7) | 9.08E+04  (±1.68E+05; n=7) | 6.59E+05  (±8.59E+05; n=7) | 5.91E+04  (±6.37E+04; n=7) | 6.37E+04  (±1.22E+05; n=7) |
|  |  | 75 – 100 | 1.33E+06  (±1.37E+06; n=7) | 8.58E+04  (±9.59E+04; n=7) | 5.80E+05  (±4.73E+05; n=7) | 4.86E+04  (±2.97E+04; n=7) | 4.28E+04  (±3.83E+04; n=7) |
| 6b | | 0 – 25 | 1.20E+08  (±9.38E+07; n=7) | 2.95E+07  (±3.36E+07; n=7) | 3.51E+07  (±3.62E+07; n=7) | 6.54E+06  (±5.19E+06; n=7) | 3.45E+06  (±2.02E+06; n=7) |
|  |  | 25 – 50 | 9.44E+07  (±6.43E+07; n=7) | 3.85E+06  (±1.70E+06; n=7) | 1.06E+07  (±1.19E+06; n=7) | 2.11E+06  (±8.63E+05; n=7) | 1.22E+06  (±4.98E+05; n=7) |
|  |  | 50 – 75 | 5.72E+06  (±1.02E+07; n=7) | 1.02E+06  (±2.14E+06; n=7) | 1.14E+06  (±1.70E+06; n=7) | 2.21E+05  (±3.28E+05; n=7) | 1.95E+05  (±3.13E+05; n=7) |
|  |  | 75 – 100 | 1.31E+06  (±1.28E+06; n=7) | 1.97E+05  (±3.94E+05; n=7) | 4.80E+05  (±2.68E+05; n=7) | 5.30E+04  (±4.98E+04; n=7) | 3.61E+04  (±5.16E+04; n=7) |
| 7 | | 0 – 25 | 1.45E+09  (±2.29E+09; n=7) | 2.18E+09  (±3.81E+09; n=7) | 5.05E+08  (±5.16E+08; n=7) | 1.16E+09  (±1.88E+09; n=7) | 5.04E+09  (±7.51E+09; n=7) |
|  |  | 25 – 50 | 8.26E+09  (±1.1E+10; n=8) | 2.56E+09  (±3.14E+09; n=8) | 1.83E+09  (±2.34E+09; n=8) | 1.11E+09  (±1.09E+09; n=8) | 9.84E+09  (±1.62E+10; n=8) |
|  |  | 50 – 75 | 2.86E+09  (±4.38E+09; n=8) | 1.14E+09  (±2.06E+09; n=8) | 1.26E+09  (±1.93E+09; n=8) | 8.22E+08  (±1.51E+09; n=8) | 5.65E+09  (±1.32E+10; n=8) |
|  |  | 75 – 100 | 7.04E+08  (±1.75E+09; n=8) | 1.79E+08  (±4.08E+08; n=8) | 1.13E+09  (±2.21E+09; n=8) | 2.80E+08  (±5.46E+08; n=8) | 1.40E+09  (±1.8E+09; n=8) |
| 8 | | 0 – 25 | 1,76E+08  (±1,98E+08; n=6) | 3,47E+08  (±3,88E+08; n=6) | 2,15E+07  (±2,00E+07; n=6) | 6,00E+06  (±5,08E+06; n=6) | 1,70E+07  (±1,74E+07; n=6) |
|  |  | 25 – 50 | 2,28E+08  (±1,70E+08; n=6) | 2,12E+08  (±1,77E+08; n=6) | 1,98E+07  (±1,60E+07; n=6) | 5,51E+06  (±4,45E+06; n=6) | 1,37E+07  (±1,16E+07; n=6) |
|  |  | 50 – 75 | 2,02E+07  (±1,92E+07; n=6) | 1,88E+07  (±2,87E+07; n=6) | 3,10E+06  (±3,02E+06; n=6) | 4,60E+05  (±3,71E+05; n=6) | 1,22E+06  (±1,19E+06; n=6) |
|  |  | 75 – 100 | 8,89E+06  (±1,60E+07; n=6) | 4,62E+06  (±6,58E+06; n=6) | 2,91E+06  (±4,16E+06; n=6) | 2,90E+05  (±3,51E+05; n=6) | 6,47E+05  (±7,94E+05; n=6) |
