# Supplementary material for: The distribution of functional N-cycle related genes and ammonia and nitrate nitrogen in soil profiles fertilized with mineral and organic N fertilizer
Source: PLoS One. 2020 Jun 2;15(6):e0228364. doi: 10.1371/journal.pone.0228364 (PMC7266355; doi:10.1371/journal.pone.0228364)
Supplement: S6 Table — The test was performed using the data from all the regularly fertilized soils (1–6). The data used refer to the whole profile of the analysed soil (0–100 cm). Numbers in table indicate the r correlation coefficients. (DOCX) [file pone.0228364.s007.docx]

**S6 Table. Pearson correlation matrix between gene copies in regularly fertilized soils.** The test was performed using the data from all the regularly fertilized soils (1-6). The data used refer to the whole profile of the analysed soil (0-100 cm). Numbers in table indicate the r correlation coefficients.

** indicates a p value <0.01, *n*=252.

|  | ***amoA***  **Archaea**  **0-100 cm** | ***amoA* Eubacteria**  **0-100 cm** | ***nifH***  **0-100 cm** | ***nirK***  **0-100 cm** | ***nosZ***  **0-100 cm** |
| --- | --- | --- | --- | --- | --- |
| ***amoA***  **archaea**  **0-100 cm** |  |  |  |  |  |
| ***amoA***  **Eubacteria**  **0-100 cm** | **0.722**** |  |  |  |  |
| ***nifH***  **0-100 cm** | **0.747**** | **0.649**** |  |  |  |
| ***nirK***  **0-100 cm** | **0.695**** | **0.9**** | **0.569**** |  |  |
| ***nosZ***  **0-100 cm** | **0.795**** | **0.858**** | **0.799**** | **0.767**** |  |
