# Supplementary material for: The distribution of functional N-cycle related genes and ammonia and nitrate nitrogen in soil profiles fertilized with mineral and organic N fertilizer
Source: PLoS One. 2020 Jun 2;15(6):e0228364. doi: 10.1371/journal.pone.0228364 (PMC7266355; doi:10.1371/journal.pone.0228364)
Supplement: S7 Table — The test was performed using the data from all the regularly fertilized farms (1–6). Numbers in table indicate the r correlation coefficients. (DOCX) [file pone.0228364.s008.docx]

**S7 Table. Pearson correlation matrix.** The test was performed using the data from all the regularly fertilized farms (1-6). Numbers in table indicate the r correlation coefficients.

|  | ***amoA* Archaea**  **0-50 cm** | ***amoA* Eubacteria 0-50 cm** | ***nifH***  **0-50 cm** | ***nirK***  **0-50 cm** | ***nosZ***  **0-50 cm** | **N-NO_3_**  **0-50 cm** | **N-NO_3_**  **75-100 cm** | **N-NH_4_**  **0-50 cm** | **N dosed y^-1^** | **Sand**  **0-50 cm** | **Silt**  **0-50 cm** | **Clay**  **0-50 cm** | **pH**  **0-50 cm** |
| --- | --- | --- | --- | --- | --- | --- | --- | --- | --- | --- | --- | --- | --- |
| ***amoA* Archaea**  **0-50 cm** |  |  |  |  |  |  |  |  |  |  |  |  |  |
| ***amoA* Eubacteria**  **0-50 cm** | **0,981*** |  |  |  |  |  |  |  |  |  |  |  |  |
| ***nifH***  **0-50 cm** | **0,962*** | **0,977*** |  |  |  |  |  |  |  |  |  |  |  |
| ***nirK***  **0-50 cm** | **0,907*** | **0,939*** | **0,847*** |  |  |  |  |  |  |  |  |  |  |
| ***nosZ***  **0-50 cm** | **0,975*** | **0,995*** | **0,992*** | **0,907*** |  |  |  |  |  |  |  |  |  |
| **N-NO_3_**  **0-50 cm** | 0,580 | **0,635*** | 0,521 | **0,787*** | 0,599 |  |  |  |  |  |  |  |  |
| **N-NO_3_**  **75-100 cm** | **0,757*** | **0,805*** | **0,679*** | **0,895*** | **0,751*** | **0,684*** |  |  |  |  |  |  |  |
| **N-NH_4_**  **0-50 cm** | **0,742*** | **0,756*** | **0,660*** | **0,809*** | **0,711*** | 0,444 | **0,837*** |  |  |  |  |  |  |
| **N**  **Dosed y^-1^** | 0,203 | 0,237 | 0,284 | 0,120 | 0,253 | -0,377 | 0,171 | 0,470 |  |  |  |  |  |
| **Sand**  **0-50 cm** | 0,540 | 0,581 | 0,539 | 0,536 | 0,553 | 0,311 | **0,718*** | **0,717*** | 0,286 |  |  |  |  |
| **Silt**  **0-50 cm** | -0,405 | -0,483 | -0,526 | -0,328 | -0,494 | -0,026 | -0,486 | -0,534 | -0,583 | **-0,860*** |  |  |  |
| **Clay**  **0-50 cm** | -0,473 | -0,444 | -0,307 | -0,568 | -0,376 | -0,546 | **-0,698*** | **-0,638*** | 0,238 | **-0,729*** | 0,277 |  |  |
| **pH**  **0-50 cm** | -0,585 | **-0,633*** | -0,591 | -0,577 | -0,608 | -0,292 | **-0,799*** | **-0,645*** | -0,358 | **-0,895*** | **0,820*** | 0,581 |  |

* indicates a *P* value < 0.05; n=252
