# Supplementary material for: The distribution of functional N-cycle related genes and ammonia and nitrate nitrogen in soil profiles fertilized with mineral and organic N fertilizer
Source: PLoS One. 2020 Jun 2;15(6):e0228364. doi: 10.1371/journal.pone.0228364 (PMC7266355; doi:10.1371/journal.pone.0228364)
Supplement: S8 Table — The test was performed using the data from all the soils 7 and 8. The data used refer to the whole profile of the analysed soil (0–100 cm). Numbers in table indicate the r correlation coefficients. (DOCX) [file pone.0228364.s009.docx]

**S8 Table. Pearson correlation matrix between gene copies in soils fertilized with a N excess.** The test was performed using the data from all the soils 7 and 8. The data used refer to the whole profile of the analysed soil (0-100 cm). Numbers in table indicate the r correlation coefficients.

|  | **SOIL 7** | | | | |
| --- | --- | --- | --- | --- | --- |
|  | ***amoA***  **Archaea**  **0-100 cm** | ***amoA* Eubacteria**  **0-100 cm** | ***nifH***  **0-100 cm** | ***nirK***  **0-100 cm** | ***nosZ***  **0-100 cm** |
| ***amoA***  **archaea**  **0-100 cm** |  |  |  |  |  |
| ***amoA***  **Eubacteria**  **0-100 cm** | **0.507**** |  |  |  |  |
| ***nifH***  **0-100 cm** | **0.617**** | 0.237 |  |  |  |
| ***nirK***  **0-100 cm** | **0.397*** | **0.926**** | 0.133 |  |  |
| ***nosZ***  **0-100 cm** | **0.729**** | **0.810**** | **0.485**** | **0.726**** |  |
|  | **SOIL 8** | | | | |
|  | ***amoA***  **Archaea**  **0-100 cm** | ***amoA* Eubacteria**  **0-100 cm** | ***nifH***  **0-100 cm** | ***nirK***  **0-100 cm** | ***nosZ***  **0-100 cm** |
| ***amoA***  **archaea**  **0-100 cm** |  |  |  |  |  |
| ***amoA***  **Eubacteria**  **0-100 cm** | **0.832**** |  |  |  |  |
| ***nifH***  **0-100 cm** | 0.270 | **0.554**** |  |  |  |
| ***nirK***  **0-100 cm** | -0.026 | 0.141 | **0.825**** |  |  |
| ***nosZ***  **0-100 cm** | -0.062 | 0.103 | **0.806**** | **0.999**** |  |

* indicates a p value <0.05; ** indicates a p value <0.01. n=32 (soil 7) and 24 (soil 8).
