# Supplementary material for: The distribution of functional N-cycle related genes and ammonia and nitrate nitrogen in soil profiles fertilized with mineral and organic N fertilizer
Source: PLoS One. 2020 Jun 2;15(6):e0228364. doi: 10.1371/journal.pone.0228364 (PMC7266355; doi:10.1371/journal.pone.0228364)
Supplement: S9 Table — Regression for nitrate content in soils (75–100 cm) (n = 10) vs. agronomic, chemical, physical, biological and meteorological data (parameters = 26). In the table are reported variables included in regressions and their importance. (DOCX) [file pone.0228364.s010.docx]

**S9 Table. PLS regression.** Regression for nitrate content in soils (75-100 cm) (*n* = 10) vs. agronomical, chemical, physical, biological and meteorological data (parameters = 26). In the table are reported variables included into regression and their importance.

| **Predictor variables** | **Importance of the predictor** | **R^2^** | **R^2^cv** |
| --- | --- | --- | --- |
| amoA archaea 0-25 | 0.11 | 0.96 | 0.95 |
| amoA eubacteria 0-25 | 0.12 |  |  |
| nirK 0-25 | 0.12 |  |  |
| Znos 0-25 | 0.12 |  |  |
| Sand 25-50 | 0.1 |  |  |
| Silt 25-50 | -0.08 |  |  |
| Clay 0-25 | -0.1 |  |  |
| pH 0-25 | -0.1 |  |  |
| pH 25-50 | -0.11 |  |  |
| NO_3_^-^ 0-25 | 0.08 |  |  |
| NH_4_^+^ 0-25 | 0.06 |  |  |
| NH_4_^+^ 25-50 | 0.11 |  |  |
